# Construction of an interactive online phytoplasma classification tool, *i*PhyClassifier, and its application in analysis of the peach X-disease phytoplasma group (16SrIII)

**DOI:** 10.1099/ijs.0.010249-0

**Published:** 2009-10

**Authors:** Yan Zhao, Wei Wei, Ing-Ming Lee, Jonathan Shao, Xiaobing Suo, Robert E. Davis

**Affiliations:** Molecular Plant Pathology Laboratory, USDA-Agricultural Research Service, Beltsville, MD 20705, USA

## Abstract

Phytoplasmas, the causal agents of numerous plant diseases, are insect-vector-transmitted, cell-wall-less bacteria descended from ancestral low-G+C-content Gram-positive bacteria in the *Bacillus*–*Clostridium* group. Despite their monophyletic origin, widely divergent phytoplasma lineages have evolved in adaptation to specific ecological niches. Classification and taxonomic assignment of phytoplasmas have been based primarily on molecular analysis of 16S rRNA gene sequences because of the inaccessibility of measurable phenotypic characters suitable for conventional microbial characterization. In the present study, an interactive online tool, *i*PhyClassifier, was developed to expand the efficacy and capacity of the current 16S rRNA gene sequence-based phytoplasma classification system. *i*PhyClassifier performs sequence similarity analysis, simulates laboratory restriction enzyme digestions and subsequent gel electrophoresis and generates virtual restriction fragment length polymorphism (RFLP) profiles. Based on calculated RFLP pattern similarity coefficients and overall sequence similarity scores, *i*PhyClassifier makes instant suggestions on tentative phytoplasma 16Sr group/subgroup classification status and ‘*Candidatus* Phytoplasma’ species assignment. Using *i*PhyClassifier, we revised and updated the classification of strains affiliated with the peach X-disease phytoplasma group. The online tool can be accessed at http://www.ba.ars.usda.gov/data/mppl/iPhyClassifier.html.

Phytoplasmas are small, insect-transmitted, cell-wall-less bacteria that cause numerous diseases in economically and environmentally important plant species worldwide (McCoy *et al.*, 1989; Lee *et al.*, 2000; Seemüller *et al.*, 2002; Hogenhout *et al.*, 2008). In infected plants, phytoplasmas colonize enucleate sieve cells of phloem tissue and induce various systemic symptoms including yellowing, shoot proliferation, witches'-broom growth, phyllody and virescence. Phylogenetic studies suggest that extant phytoplasmas share a common evolutionary root and are descended from low-G+C-content Gram-positive bacteria in the *Bacillus*–*Clostridium* group (Weisburg *et al.*, 1989; Gundersen *et al.*, 1994; Sears & Kirkpatrick, 1994; Zhao *et al.*, 2005; Wei *et al.*, 2007). After evolutionary divergence from an *Acholeplasma*-like last common ancestor, phytoplasmas emerged as a discrete clade and a large number of widely divergent phytoplasma lineages have evolved in adaptation to a broad range of bio- and geo-ecological niches (Lee *et al.*, 1992a, b, 1993a, 2000; Marcone *et al.*, 1999; Davis *et al.*, 2005; Martini *et al.*, 2007; Wei *et al.*, 2007, 2008a, b; Cai *et al.*, 2008). Since phytoplasmas cannot yet be cultured successfully in a cell-free medium and measurable phenotypic characters suitable for conventional microbial characterization remain inaccessible, current knowledge on biodiversity and genetic interrelationships among phytoplasma strains has been derived mainly from aetiological studies, applications of serological and nucleic acid-based assay techniques and molecular analysis of evolutionarily conserved gene sequences.

As in other prokaryotes (Rosselló-Mora & Amann, 2001), genes encoding 16S rRNAs are highly conserved across the phytoplasma clade yet contain ample information for differentiation of diverse phytoplasma strains. The establishment of a 16S rRNA gene sequence-based restriction fragment length polymorphism (RFLP) profiling scheme (Lee *et al.*, 1993b; Gundersen *et al.*, 1994), with periodic updates (Lee *et al.*, 1998, 2000, 2004a, b), has provided reliable molecular markers for identification and classification of a broad array of phytoplasmas into a system of groups and subgroups, with each group containing at least one distinct phytoplasma species and each subgroup containing strains with identical or nearly identical RFLP patterns. The availability of this classification scheme greatly stimulated and expanded phytoplasma research during the past decade and, as a result, novel phytoplasma lineages have been discovered at an increasingly rapid pace in emerging diseases throughout the world. Sequence information held in 16S rRNA genes has also served as a baseline for ‘*Candidatus* Phytoplasma’ candidate species delineation (referred to here as ‘*Ca.* Phytoplasma’ species, although the designation *Candidatus* is not covered by the Bacteriological Code and such names therefore have no standing in nomenclature) as recommended by the Phytoplasma Taxonomy Group of the International Research Program on Comparative Mycoplasmology (IRPCM Phytoplasma/Spiroplasma Working Team – Phytoplasma taxonomy group, 2004). So far, 19 phytoplasma 16S rRNA gene RFLP (16Sr) groups have been delineated on the basis of actual enzymic RFLP/gel electrophoretic analysis of PCR-amplified 16S rRNA gene fragments (Gundersen *et al.*, 1994; Lee *et al.*, 1998, 2000, 2004a, b; Al-Saady *et al.*, 2008), and 27 ‘*Ca.* Phytoplasma’ species have been formally described (IRPCM Phytoplasma/Spiroplasma Working Team – Phytoplasma taxonomy group, 2004; Firrao *et al.*, 2005; Lee *et al.*, 2006; Valiunas *et al.*, 2006; Arocha *et al.*, 2007; Al-Saady *et al.*, 2008).

Recently, development and application of computer programs for virtual RFLP analysis have made possible high-throughput differentiation and identification of phytoplasma strains (Wei *et al.*, 2007, 2008b; Cai *et al.*, 2008). By mimicking actual ‘wet’ laboratory restriction enzyme digestions and subsequent gel electrophoresis, computerized RFLP pattern comparisons and similarity coefficient calculations identified ten novel phytoplasma 16Sr groups and dozens of new subgroup lineages (Wei *et al.*, 2007, 2008b; Cai *et al.*, 2008; Quaglino *et al.*, 2009), significantly expanding the existing 16S rRNA gene RFLP-based classification scheme. Several potentially novel ‘*Ca.* Phytoplasma’ species were also suggested from such computer-aided analysis (Wei *et al.*, 2007). Since the applicability and extended potential of virtual RFLP analysis have been evident in delineation of novel phytoplasma groups and subgroups, in elucidating candidates for novel species descriptions and in routine identification of phytoplasma strains, development of a user-friendly platform for streamlined virtual RFLP analysis is highly desired and is expected by the phytoplasma research community.

In the present study, we devised an interactive online tool, *i*PhyClassifier, for real-time identification and classification of phytoplasmas. Besides implementing the concepts and programs that we described previously (Wei *et al.*, 2007, 2008b), *i*PhyClassifier integrates additional functions that we developed in the present study. Such new functions include overall sequence comparison and similarity score calculation, intelligent trimming of input sequences and publication-ready virtual gel plotting. The newly developed virtual gel-plotting function is able to generate not only virtual gel images resulting from multiple enzyme analysis of a single 16S F2nR2 DNA sequence from any given strain but also virtual gel images resulting from a single enzyme digestion of multiple DNA sequences. *i*PhyClassifier also incorporates carefully curated databases of phytoplasma 16S rRNA gene sequences for up-to-date classification and comparative studies. A simple operation of *i*PhyClassifier on a user input sequence can quickly lead to identification of the phytoplasma strain under study, providing suggestions on its tentative 16Sr group/subgroup classification status and ‘*Ca.* Phytoplasma’ species (or related strain) assignment. As a case study for *i*PhyClassifier application, we revised and updated the classification status of phytoplasma strains affiliated with the peach X-disease phytoplasma group.

## Program components

The current version of *i*PhyClassifier contains the following three program modules: a sequence similarity search and pairwise sequence similarity score calculation module (PM1), an intelligent sequence trimming and virtual RFLP analysis module (PM2) and a virtual electrophoresis gel image plotting module (PM3).

PM1 carries out two functions. Firstly, it performs pairwise nucleotide sequence comparisons (query against database entries) using the basic local alignment search tool (blast; Altschul *et al.*, 1990) to identify a query's phylogenetically close neighbours quickly and to determine whether or not a query sequence is of phytoplasma origin. Secondly, PM1 creates a global sequence alignment between the query sequence and sequences from the reference strain of each known ‘*Ca.* Phytoplasma’ species using the clustal w algorithm (Thompson *et al.*, 1994) and calculates percentage nucleotide sequence similarity scores using the Myers–Miller algorithm (Myers & Miller, 1988).

PM2 consists of two Perl scripts, TrimF2nR2 and RFLP_pattern_comparison. The TrimF2nR2 script, developed in the present study, prepares input nucleotide sequences for simulated enzymic digestions. The script parses through input sequences for generic annealing sites of phytoplasmal universal primers R16F2n and R16R2 (Gundersen & Lee, 1996) and trims each input sequence to the full-length F2nR2 region, which includes the primer annealing sites (Wei *et al.*, 2007). On each trimmed F2nR2 sequence, the RFLP_pattern_comparison script conducts simulated enzymic digestions, records the length of each restriction fragment and performs pairwise comparisons of the recorded fragment lengths. Based on summarized numbers of similar and dissimilar fragments, the script calculates a similarity coefficient (*F*) for each pair of phytoplasma strains, as described previously (Wei *et al.*, 2008b).

PM3 consists of two Perl scripts, VGelME and VGelMS; both were developed in the present study. While VGelME generates virtual electrophoresis gel images resulting from *in silico* digestions of a single input sequence (an F2nR2 fragment from a single phytoplasma strain) by 17 individual enzymes, VGelMS produces gel images resulting from *in silico* digestions of multiple input sequences (F2nR2 fragments from multiple phytoplasma strains) by a single restriction enzyme. The latter helps to identify key restriction enzymes that distinguish different group and subgroup patterns.

## 16S rRNA gene sequence databases

The current version of *i*PhyClassifier incorporates three 16S rRNA gene sequence databases: DB1, a set of full- or near-full-length 16S rRNA gene sequences from reference strains of all formally described ‘*Ca.* Phytoplasma’ species, reference strains of ‘*Ca.* Phytoplasma’ species that were proposed by the IRPCM Phytoplasma/Spiroplasma Working Team – Phytoplasma taxonomy group (2004) but have not yet been formally described, reference strains of potentially novel ‘*Ca.* Phytoplasma’ species identified in our previous study (Wei *et al.*, 2007) and all type strains of named prokaryotic species; DB2, a set of F2nR2 sequences from representative strains of established phytoplasma 16Sr groups and subgroups; and DB3, a set of F2nR2 sequences compiled from all phytoplasma 16S rRNA gene sequences currently deposited in GenBank, EMBL and DDBJ. The names of the non-phytoplasmal prokaryotic species in DB1 are those validly published in the *International Journal of Systematic and Evolutionary Microbiology* (formerly *International Journal of Systematic Bacteriology*) and were obtained from the List of Prokaryote Names with Standing in Nomenclature (http://www.bacterio.cict.fr/validationlists.html; Euzéby, 1997) (last update 9 October 2008). All 16S rRNA gene sequences were downloaded from the NCBI nucleotide sequence database at http://www.ncbi.nlm.nih.gov/gquery/gquery.fcgi using the Entrez search and retrieval tool (Wheeler *et al.*, 2005). All databases are maintained using MySQL.

## Operational process

**Fig. 1. f1:**
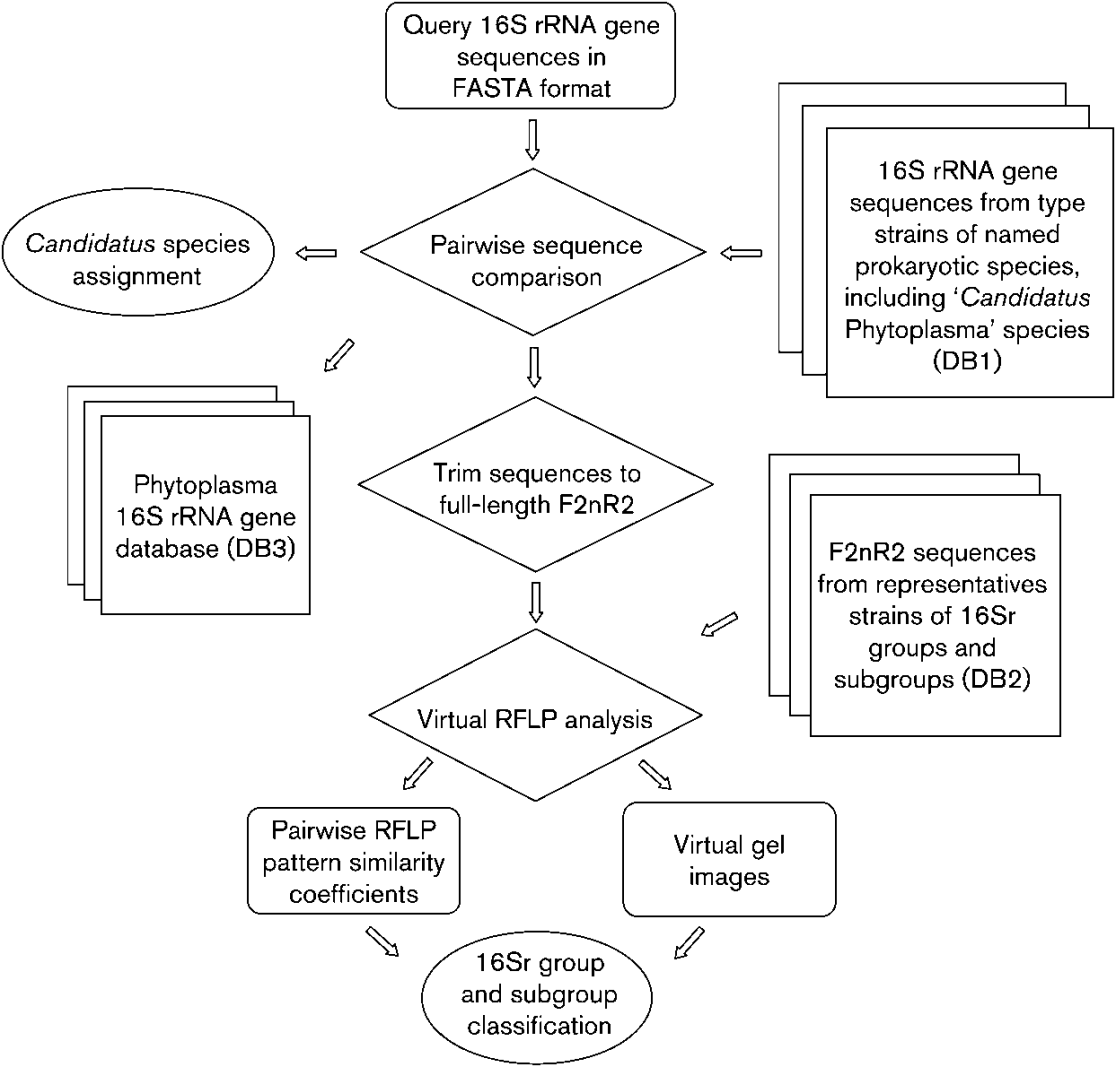
Diagrammatic representation of the operational process of *i*PhyClassifier. Rectangles represent input and output files, squares represent databases, diamonds represent computational operations and ovals represent recommendations on tentative 16Sr group/subgroup classification status and ‘*Ca.* Phytoplasma’ species assignment.

**Fig. 2. f2:**
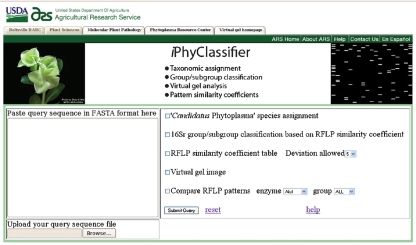
Screenshot of the *i*PhyClassifier web page.

The overall operational process of *i*PhyClassifier is outlined in Fig. 1. The aim of the entire operation is to provide meaningful suggestions on tentative 16Sr group/subgroup classification status and ‘*Ca.* Phytoplasma’ species (or related strain) assignment for any phytoplasma strain under study. The operation starts by receiving a query sequence(s) from the user. The queries, in fasta format, can be either uploaded as a precompiled file from the user's computer to the *i*PhyClassifier web server or directly typed or pasted into the query sequence input window in the *i*PhyClassifier web page (Fig. 2; http://www.ba.ars.usda.gov/data/mppl/iPhyClassifier.html).

The first step of the *i*PhyClassifier operation is to perform pairwise comparisons between each query sequence and the sequences in database DB1 for quick identification of the query's phylogenetically close neighbours. In this initial stage of sequence comparison, the blast algorithm is used. If none of the ‘*Ca.* Phytoplasma’ species in DB1 appears among the top 50 hits returned from the blast search, or one or more ‘*Ca.* Phytoplasma’ species appears among the top hits but shares ≤91 % sequence similarity with the query, the operation will abort, warning that the query sequence is unlikely to originate from a phytoplasma. If at least one ‘*Ca.* Phytoplasma’ species is among the top hits returned from the blast search and shares ≥92 % sequence similarity with the query, the operation will proceed and the query sequence will be fed into the clustal w program for global alignment with all phytoplasma sequences in the database DB1Phy (a subset of DB1) and for sequence similarity score calculation. Such a combined search strategy aids identification of the query's phylogenetically closest neighbour with a significantly reduced computing time (Chun *et al.*, 2007). In accordance with the convention on 16S rRNA gene sequence-based prokaryotic species delineation (Murray & Schleifer, 1994; Stackebrandt & Goebel, 1994), *i*PhyClassifier implements the recommendation of the IRPCM Phytoplasma/Spiroplasma Working Team – Phytoplasma taxonomy group (2004) and presets 97.5 % 16S rRNA gene sequence similarity as the cut-off value for novel ‘*Ca.* Phytoplasma’ species recognition. Since the generally conserved 16S rRNA gene sequence contains pockets of hypervariable regions, the sequence similarity score calculation should be based upon comparison of full- or near-full-length 16S rRNA gene sequences. It requires that each query sequence covers at least 1200 positions within a 16S rRNA gene. The output of this operational step consists of the assignment of the query strain tentatively to an existing ‘*Ca.* Phytoplasma’ species as a related strain or the suggestion that the query represents a potentially novel ‘*Ca.* Phytoplasma’ species, depending on the sequence similarity scores.

The second step of the *i*PhyClassifier operation is to trim each query sequence to the full-length F2nR2 region using regular expressions that match primer pair R16F2n/R16R2. This step is critical because, in the 16S rRNA gene-based phytoplasma classification scheme, strains are classified into groups and subgroups based strictly on RFLP patterns derived from 16S rRNA gene F2nR2 fragments (Lee *et al.*, 1998, 2000; Wei *et al.*, 2007, 2008b).

The third step of *i*PhyClassifier operation is to simulate restriction digestions on trimmed F2nR2 fragments, compare the RFLP pattern types derived from each query strain to those derived from database DB2 and calculate pairwise RFLP pattern similarity coefficients. In this step, *i*PhyClassifier implements the criterion proposed in our previous work (Wei *et al.*, 2008b), presetting 0.97 as the threshold similarity coefficient for delineation of a new subgroup RFLP pattern type within a given group. Thus, if the virtual F2nR2 RFLP pattern derived from a 16S rRNA gene of a phytoplasma strain under study has a similarity coefficient of 0.97 or less with 16S rRNA genes of all existing representative or reference strains of the given group, a new subgroup pattern type is recognized. Adoption of 0.97 as the threshold similarity coefficient for new subgroup delineation is warranted because it reflects precisely the existing subgroup classification scheme, in which as few as one restriction site difference can distinguish a new subgroup. A similarity coefficient of 0.85 or less with all previously recognized subgroups signals that the strain under study may represent a new 16Sr group, in agreement with all previously designated groups. RFLP patterns that have a similarity coefficient of 0.99 or 0.98 with the standard pattern type of the designated representative or reference member in a given subgroup are considered as variants of the standard pattern type. These variants or minor pattern types are denoted with one or two asterisks (* or **) following the corresponding subgroup letter, for example 16SrI-A* (*F*=0.99) and 16SrI-A** (*F*=0.98), as suggested previously (Wei *et al.*, 2008b). Since similarity coefficient values are influenced by both the number and the particular set of restriction enzymes selected for RFLP analysis, the threshold similarity coefficients for new subgroup and group pattern type delineations are based strictly on the use of a specific set of 17 restriction enzymes originally established for classification of phytoplasmas using actual gel electrophoresis-based RFLP analysis (Lee *et al.*, 1998). The output of this operational step is the assignment of the strain under study into an existing subgroup or erection of a new subgroup. Because the presence of two heterogeneous *rrn* operons in individual phytoplasma strains is widespread (see the section on interoperon sequence heterogeneity below), final subgroup designation of strains with heterogeneous *rrn* operons should be based on composite patterns derived from both *rrn* operons. At the end of this operational step, the query sequence is added to database DB3.

Concomitant with similarity coefficient calculation, which generates numerical output of the RFLP pattern analysis, the *i*PhyClassifier also provides visual output, i.e. virtual gel images resulting from the RFLP pattern analysis. The gel images reveal informative sites or ‘visible’ genetic markers along the 16S rRNA gene sequences, transforming sequence information into accessible ‘virtual phenotypic characters’ for phytoplasma strain differentiation and classification.

## Critical issues

Since the operation of *i*PhyClassifier is solely dependent upon sequence information, any error in a query (input) sequence that misrepresents the phytoplasma strain under study could result in erroneous group/subgroup classification and ‘*Ca.* Phytoplasma’ species assignment. While sequence errors may arise at various stages during PCR amplification, plasmid multiplication and DNA sequencing, they usually occur randomly and can be rectified by sample replications. To ensure credible operations of *i*PhyClassifier, we highly recommend that consistent sequence data be obtained from at least two independent samples, i.e. from two or more infected plants or insect individuals. If only one infected plant or insect sample is available for study, consistent sequence data from at least two independently cloned DNA segments derived from two separate PCRs must be obtained. Each clone (plasmid) should be sequenced in both directions and a minimum of triplicate coverage per base position achieved.

The genomes of all four completely sequenced phytoplasma strains and numerous reference strains of ‘*Ca.* Phytoplasma’ species harbour two rRNA operons, *rrnA* and *rrnB* (IRPCM Phytoplasma/Spiroplasma Working Team – Phytoplasma taxonomy group, 2004; Oshima *et al.*, 2004; Bai *et al.*, 2006; Kube *et al.*, 2008; Tran-Nguyen *et al.*, 2008). In many strains, the sequences of the two *rrn* operons differ (Lee *et al.*, 1993b, 1998; Firrao *et al.*, 1996; Liefting *et al.*, 1996; Davis & Sinclair, 1998; Jomantiene *et al.*, 2002; Davis *et al.*, 2003). For those phytoplasma strains with two heterogeneous *rrn* operons, if the sequence variations between the two operons fall within restriction sites within the 16S rRNA gene F2nR2 region, two different virtual 16Sr RFLP pattern types will result from *i*PhyClassifier operation. It is therefore important to distinguish between subgroup pattern types and final subgroup designation and to avoid erroneous assignment of the same strain into two different 16Sr subgroups. In this regard, *i*PhyClassifier adopts the recommendation of Wei *et al.* (2008b) and uses a three-letter subgroup designation, where the first and second letters (in parentheses) denote the RFLP pattern types of *rrnA* and *rrnB*, respectively, and the third letter designates the 16Sr subgroup. For example, paulownia witches'-broom (PaWB) phytoplasma, a member of the previously delineated subgroup 16SrI-D (Lee *et al.*, 1998), possesses two sequence-heterogeneous rRNA operons that display different 16Sr RFLP patterns, characteristic of subgroups 16SrI-B and 16SrI-D, respectively (Wei *et al.*, 2008b); therefore, the subgroup status of PaWB is redesignated 16SrI-(B/D)D.

## An update of peach X-disease phytoplasma group (16SrIII) classification

X-disease of peach in North America was first reported in the early 1930s (Stoddard, 1934); however, it wasn't until the 1970s that the causal agent of the disease was identified to be a phytoplasma (Granett & Gilmer, 1971; Macbeath *et al.*, 1972). Since then, numerous phytoplasmas affecting stone-fruit trees, as well as many other plants, were found to be closely related to the phytoplasma(s) associated with X-disease of peach in the western and eastern USA. On the basis of RFLP analyses of 16S rRNA gene sequences, these strains have been classified into a single RFLP group, 16SrIII (Lee *et al.*, 1993b, 1998; Jomantiene *et al.*, 2002). This group includes strains that differ in their geographical origin, plant host/insect vector relations and symptoms induced in infected plants. It has been found that such varied strains may carry distinct molecular markers in their 16S rRNA gene sequences (Lee *et al.*, 1993b, 1998). Based on actual enzymic RFLP analysis of 16S rRNA genes F2nR2 fragments, 14 subgroups were delineated (Lee *et al.*, 1993b, 1998; Jomantiene *et al.*, 2002), each subgroup consisting of strains that share identical or nearly identical RFLP patterns.

**Fig. 3. f3:**
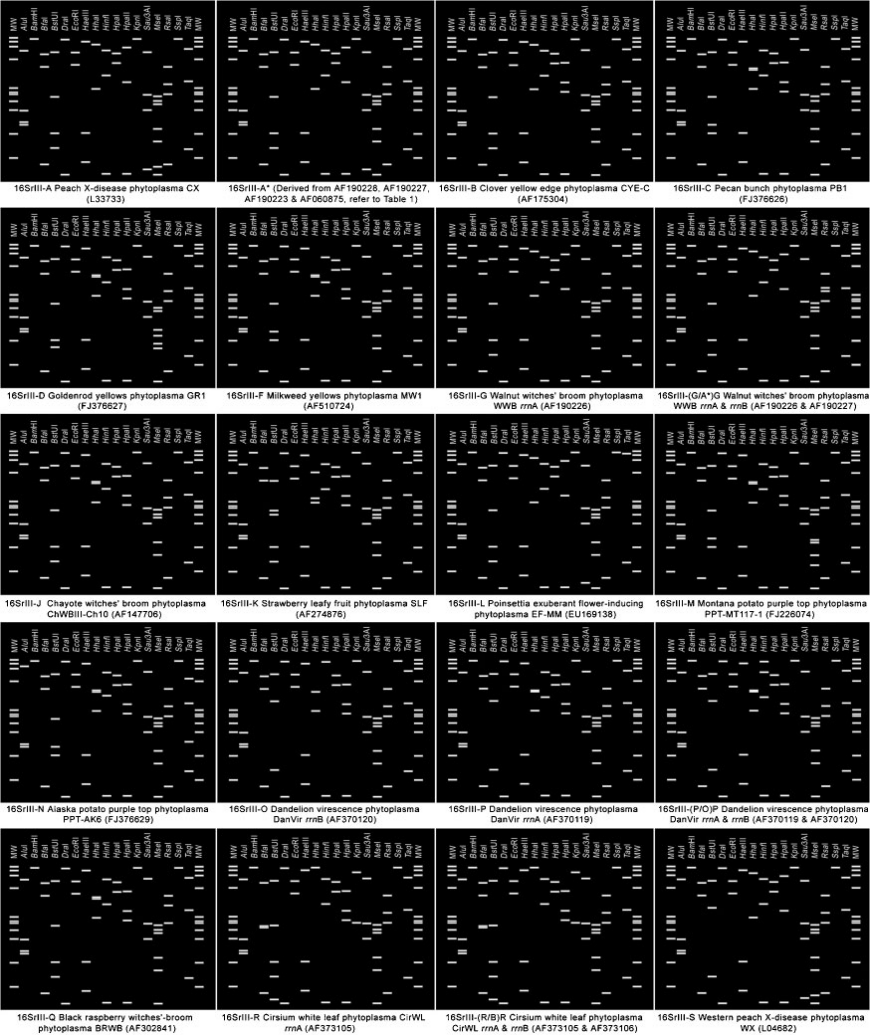
Virtual RFLP patterns derived from *in silico* digestions of 16S rRNA gene F2nR2 fragments from representative strains of novel and previously delineated 16SrIII subgroups. Recognition sites for the following 17 restriction enzymes were used in the simulated digestions: *Alu*I, *Bam*HI, *Bfa*I, *Bst*UI (*Tha*I), *Dra*I, *Eco*RI, *Hae*III, *Hha*I, *Hin*fI, *Hpa*I, *Hpa*II, *Kpn*I, *Sau*3AI (*Mbo*I), *Mse*I, *Rsa*I, *Ssp*I and *Taq*I. Lanes MW, *φ*X174 DNA digested with *Hae*III.

In the present study, using *i*PhyClassifier, we updated the classification status of the phytoplasma strains affiliated with the peach X-disease phytoplasma group. Standard virtual 16S rRNA gene RFLP patterns were generated for representative strains of the 14 previously delineated subgroups (Table 1 and Fig. 3). Composite RFLP patterns that reflect the presence of two heterogeneous *rrn* operons in the representative strains of three subgroups, 16SrIII-(A*/G)G, 16SrIII-(O/P)P and 16SrIII-(B/R)R, were also generated (Fig. 3).

Four new 16SrIII subgroup lineages were delineated in the present study. These new subgroups include subgroup 16SrIII-L, represented by poinsettia exuberant flower-inducing phytoplasma strain EF-MM (GenBank accession no. EU169138), subgroup 16SrIII-M, represented by Montana potato purple top phytoplasma strain PPT-MT117-1 (FJ226074), and subgroup 16SrIII-N, represented by Alaska potato purple top phytoplasma strain PPT-AK6 (FJ376629) (Table 1, Fig. 3 and Supplementary Table S1, available in IJSEM Online). Just as various strains classified in a given subgroup would be expected to share common biological characteristics, strains classified in different subgroups may have distinguishing biological properties, as apparently illustrated by the newly delineated subgroups III-L, III-M and III-N. We recognize that, in many cases, distinguishing biological characteristics may currently be unknown.

Results from the current study revealed that the RFLP patterns derived from three 16S rRNA gene sequences of peach X-disease phytoplasma strains that originated from the western USA (GenBank accession nos L04682, AF533231 and EU168790) are identical and novel (Fig. 3). This novel pattern has a similarity coefficient of 0.97 with the pattern from the 16SrIII-A representative strain Canada peach X-disease phytoplasma (CX; GenBank accession no. L33733), and has similarity coefficients of less than 0.97 with the patterns from the representative strains of all other 16SrIII subgroups (Supplementary Table S1). Therefore, these western X-disease phytoplasma strains may constitute a distinct lineage within the X-disease phytoplasma group. We recommend that a new subgroup, 16SrIII-S, be erected to accommodate these strains and to designate strain WX (GenBank accession no. L04682) as the representative member of the subgroup. Previous aetiological and epidemiological studies suggested that WX and CX possessed distinct biological properties. Strain WX was prevalent in the western USA and was found to be transmitted predominantly by *Colladonus montanus*, while CX was found to be transmitted mainly by *Paraphlepsius irroratus* and to occur in eastern USA and eastern Canada (Sinha & Chiykowski, 1980; Chiykowski & Sinha, 1990; Kirkpatrick *et al.*, 1990; Liefting & Kirkpatrick, 2003). Interestingly, these distinguishing characteristics correlate with the classification of these strains into two separate subgroups in this study. Thus, the significance of subgroup delineations lies in the potential of the subgroup-level molecular markers to distinguish closely related strains that may differ in subtle but biologically significant properties.

In addition, the current study also identified a new pattern type that is a variant of standard 16SrIII-A pattern (with a similarity coefficient of 0.99). 16S rRNA gene sequences that exhibited this variant pattern (16SrIII-A*; Fig. 3) include those from phytoplasma strains that were classified in subgroups 16SrIII-E, 16SrIII-H and 16SrIII-I (Table 1 and Supplementary Table S1), indicating that these phytoplasma strains may possess two sequence-heterogeneous rRNA operons, as does the walnut witches' broom phytoplasma [16SrIII-(A*/G)G] (Supplementary Table S1).

**Fig. 4. f4:**
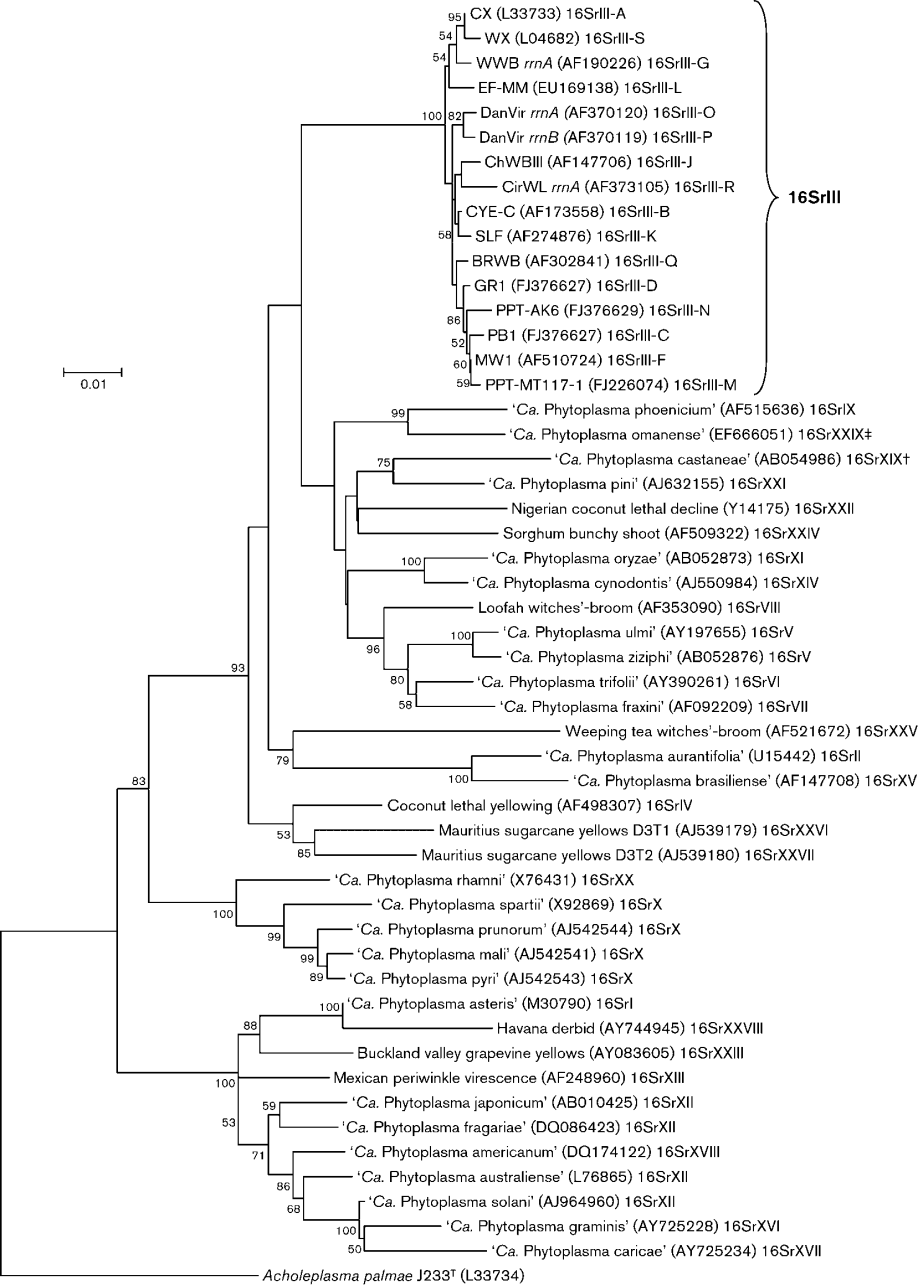
Phylogenetic tree inferred from analysis of 16S rRNA gene sequences. Minimum-evolution analysis was conducted using the close neighbour interchange (CNI) algorithm implemented in mega4 (Tamura *et al.*, 2007). The initial tree for the CNI search was obtained by the neighbour-joining method. The reliability of the analysis was subjected to a bootstrap test with 1000 replicates. The taxa used in the phylogenetic tree reconstruction included reference strains of each phytoplasma 16Sr group, reference strains of subgroups belonging to the peach X-disease phytoplasma group and reference strains of each ‘*Ca.* Phytoplasma’ species (‘*Ca*. Phytoplasma allocasuarinae’ and ‘*Ca*. Phytoplasma lycopersici’ were not included because the available 16S rRNA gene sequences did not encompass the entire F2nR2 region. Reference strains of subgroups 16SrIII-E, 16SrIII-H and 16SrIII-I were not included because complete sequence information is not available). The sequence of *Acholeplasma palmae* J233^T^ served as an outgroup during phylogenetic tree reconstruction. Bar, 0.01 nucleotide substitutions per site. †In the report by Jung *et al.* (2002), ‘*Ca*. Phytoplasma castaneae’ was assigned to group VI according to DNA sequence similarity, rather than results from RFLP analysis. In accordance with the more widely accepted RFLP-based classification system, this phytoplasma was reassigned to group 16SrXIX by Wei *et al.* (2007). ‡The original reference (Al-Saady *et al.*, 2008) reported ‘*Ca.* Phytoplasma omanense’ as the reference member of a novel group designated group 16SrXIX. However, the group number 16SrXIX had been published previously (Wei *et al.*, 2007) to accommodate a different phytoplasma, ‘*Ca*. P. castaneae’. Therefore, we assign ‘*Ca*. P. omanense’ to a new group, 16SrXXIX, subgroup 16SrXXIX-A.

**Fig. 5. f5:**
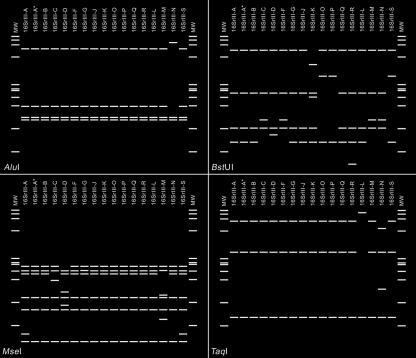
Key restriction enzymes that distinguish novel 16SrIII subgroup pattern types. Lanes MW, *φ*X174 DNA digested with *Hae*III.

The representative strains of the novel 16SrIII subgroups delineated using *i*PhyClassifier are clustered phylogenetically with representative strains of previously recognized 16SrIII subgroups, forming a subclade (Fig. 4). The tree topology indicated that clustering of 16Sr RFLP groups is consistent with 16S rRNA gene sequence-based phylogeny. However, we note that, not surprisingly, multiple strains belonging to a single 16Sr subgroup may not necessarily cluster together in a phylogenetic subtree (not shown), since subgroup classification is based upon a subset (i.e. recognition sites of 17 restriction enzymes) of the characters that are used in phylogenetic analysis. According to *i*PhyClassifier analysis, novel subgroup patterns delineated in the present study can be distinguished from previously recognized patterns by *in silico* digestions with a few key enzymes. For example, subgroup pattern 16SrIII-L can be distinguished from others by *Taq*I digestion, separation of subgroup pattern 16SrIII-M from all other subgroup pattern types can be achieved by *Mse*I digestion, distinction between subgroup pattern 16SrIII-N and other subgroup pattern types can be made by either *Alu*I or *Taq*I digestion and subgroup pattern 16SrIII-S can be differentiated from other pattern types by *Mse*I and *Bst*UI digestions (Fig. 5).

In conclusion, RFLP profiling of PCR-amplified 16S rRNA gene fragments has served as a primary means for differentiation and classification of phytoplasmas over the past 15 years. We anticipate that 16S rRNA gene RFLP patterns will continue to be exploited as molecular genetic markers for identification of known phytoplasmas and discovery of novel phytoplasmas in the foreseeable future. *i*PhyClassifier provides a user-friendly platform to identify such molecular genetic markers quickly in diverse phytoplasma strains and to transform them into virtual phenotypic characters in the form of sequence similarity scores, RFLP pattern similarity coefficients and virtual gel images followed by instant suggestions on tentative 16Sr group/subgroup classification status and ‘*Ca.* Phytoplasma’ species assignment for the phytoplasma strains under study. Rapid delineation of novel phytoplasma lineages affiliated with the peach X-disease phytoplasma group demonstrated the feasibility and effectiveness of the *i*PhyClassifier operation. In addition, since computer-generated RFLP patterns (Wei *et al.*, 2007, 2008b; Cai *et al.*, 2008; this paper) faithfully replicate the classical, authoritative patterns that had been established by conventional RFLP analysis (Lee *et al.*, 1993b, 1998), *i*PhyClassifier can serve as an assistant, by providing reference strain RFLP patterns, to researchers who prefer to perform conventional RFLP analysis for identification and classification of unknown or novel phytoplasmas. The framework of *i*PhyClassifier can easily be expanded to accommodate the full-length 16S rRNA gene, other phytoplasma genes and multilocus virtual RFLP analyses when more phytoplasma genomic DNA sequences become available.

## Supplementary Material

[Supplementary Table]

## Figures and Tables

**Table 1. t1:** Classification of phytoplasmas in the peach X-disease phytoplasma group (16SrIII) based on RFLP patterns derived from 16S rRNA gene F2nR2 sequences Novel subgroups delineated in the present study are highlighted in bold. Asterisks indicate variants of the designated subgroup or pattern type. In the Subgroup column, letters in parentheses denote the RFLP pattern types of *rrnA/rrnB*. Representative (or reference) strains are the first strains for which a given subgroup pattern type was recognized.

**Subgroup**	**Virtual pattern**	**GenBank accession no.**	**Representative strain**	**Original source**	**Notes**
16SrIII-A	16SrIII-A	L33733	Peach X-disease phytoplasma CX	Peach (Canada)	No heterogeneous *rrn* reported so far
16SrIII-B	16SrIII-B	AF175304	Clover yellow edge phytoplasma CYE-C	Clover (Canada)	No heterogeneous *rrn* reported so far
16SrIII-C	16SrIII-C	FJ376626	Pecan bunch phytoplasma PB1	Pecan (Georgia, USA)	No heterogeneous *rrn* reported so far
16SrIII-D	16SrIII-D	FJ376627	Goldenrod yellows phytoplasma GR1	Goldenrod (New York, USA)	No heterogeneous *rrn* reported so far
16SrIII-(A*/?)E	16SrIII-A*, *rrnA*	AF190228	Spiraea stunt phytoplasma SP1	Spiraea (New York, USA)	Incomplete; *rrnB* sequence unavailable
16SrIII-F	16SrIII-F	AF510724	Milkweed yellows phytoplasma MW1	Milkweed (New York, USA)	No heterogeneous *rrn* reported so far
16SrIII-(G/A*)G	16SrIII-G, *rrnA*	AF190226	Walnut witches' broom phytoplasma WWB	Walnut (Georgia, USA)	Composite pattern is available (Fig. 4)
	16SrIII-A*, *rrnB*	AF190227	Walnut witches' broom phytoplasma WWB	Walnut (Georgia, USA)	Composite pattern is available (Fig. 4)
16SrIII-(A*/?)H	16SrIII-A*, *rrnA*	AF190223	Poinsettia branch-inducing phytoplasma PoiBI	Poinsettia (USA)	Incomplete; *rrnB* sequence unavailable
16SrIII-(A*/?)I	16SrIII-A*, *rrnA*	AF060875	Virginia grapevine yellows phytoplasma VGYIII	Grapevine (Virginia, USA)	Incomplete; *rrnB* sequence unavailable
16SrIII-J	16SrIII-J	AF147706	Chayote witches' broom phytoplasma ChWBIII (Ch10)	Chayote (Brazil)	No heterogeneous *rrn* reported so far
16SrIII-K	16SrIII-K	AF274876	Strawberry leafy fruit phytoplasma SLF	Strawberry (Maryland, USA)	No heterogeneous *rrn* reported so far
**16SrIII-L**	16SrIII-L	EU169138	Poinsettia exuberant flower-inducing phytoplasma EF-MM	Poinsettia (Mexico)	No heterogeneous *rrn* reported so far
**16SrIII-M**	16SrIII-M	FJ226074	Montana potato purple top phytoplasma PPT-MT117-1	Potato (Montana, USA)	No heterogeneous *rrn* reported so far
**16SrIII-N**	16SrIII-N	FJ376629	Alaska potato purple top phytoplasma PPT-AK6	Potato (Alaska, USA)	No heterogeneous *rrn* reported so far
16SrIII-(P/O)P	16SrIII-O, *rrnB*	AF370120	Dandelion virescence phytoplasma DanVir	Dandelion (Lithuania)	Composite pattern is available (Fig. 4)
	16SrIII-P, *rrnA*	AF370119	Dandelion virescence phytoplasma DanVir	Dandelion (Lithuania)	Composite pattern is available (Fig. 4)
16SrIII-Q	16SrIII-Q	AF302841	Black raspberry witches'-broom phytoplasma BRWB	Black raspberry (Oregon, USA)	No heterogeneous *rrn* reported so far
16SrIII-(R/B)R	16SrIII-R, *rrnA*	AF373105	Cirsium white leaf phytoplasma CirWL	Cirsium (Lithuania)	Composite pattern is available (Fig. 4)
	16SrIII-B, *rrnB*	AF373106	Cirsium white leaf phytoplasma CirWL	Cirsium (Lithuania)	Composite pattern is available (Fig. 4)
**16SrIII-S**	16SrIII-S	L04682	Western peach X-disease phytoplasma WX	Peach (California, USA)	No heterogeneous *rrn* reported so far

## Abbreviations

- RFLP, restriction fragment length polymorphism
