# Supplementary material for: Construction of an interactive online phytoplasma classification tool, iPhyClassifier, and its application in analysis of the peach X-disease phytoplasma group (16SrIII)
Source: Int J Syst Evol Microbiol. 2009 Oct;59(Pt 10):2582–93. doi: 10.1099/ijs.0.010249-0 (PMC2884932; doi:10.1099/ijs.0.010249-0)
Supplement: [Supplementary Table] [file supp_59_10_2582__index.html]

 Construction of an interactive online phytoplasma classification tool, iPhyClassifier, and its application in analysis of the peach X-disease phytoplasma group (16SrIII) -- Zhao et al. 59 (10): 2582 Data Supplement - Supplementary Table -- International Journal of Systematic and Evolutionary Microbiology 

### Construction of an interactive online phytoplasma classification tool, *i*PhyClassifier, and its application in analysis of the peach X-disease phytoplasma group (16SrIII), by Y. Zhao, W. Wei, I.-M. Lee, J. Shao, X. Suo and R. E. Davis

*International Journal of Systematic and Evolutionary Microbiology* vol. **59**, part 10, pp. 2582 - 2593

**Supplementary Table S1.** Similarity coefficients derived from analysis of virtual RFLP patterns of 16S rRNA F2nR2 sequences from phytoplasma strains in the peach X-disease group (16SrIII). [MS Excel file] (54 KB)

  
  
